# Mitochondrial genome of the acorn barnacle *Tetraclita rufotincta* Pilsbry, 1916: highly conserved gene order in Tetraclitidae

**DOI:** 10.1080/23802359.2017.1413305

**Published:** 2017-12-08

**Authors:** Jun Song, Xin Shen, Ka Hou Chu, Benny Kwok Kan Chan

**Affiliations:** a Jiangsu Key Laboratory of Marine Biotechnology/Jiangsu Institute of Marine Resources, Huaihai Institute of Technology, Lianyungang, China;; b Co-Innovation Center of Jiangsu Marine Bio-industry Technology, Lianyungang, China;; c Simon F. S. Li Marine Science Laboratory, School of Life Sciences, The Chinese University of Hong Kong, Hong Kong, China;; d Biodiversity Research Center, Academia Sinica, Taipei, Taiwan

**Keywords:** *Tetraclita rufotincta*, mitochondrial genome, gene order, phylogeny

## Abstract

The complete mitochondrial genome of the intertidal barnacle *Tetraclita rufotincta* Pilsbry, 1916 (Crustacea: Maxillopoda: Sessilia) is presented. The genome is a circular molecule of 15,236 bp, which encodes a set of 37 typical metazoan mitochondrial genes. All non-coding regions are 438 bp in length, with the longest one speculated as the control region (242 bp), which is located between *srRNA* and *trnK*. Comparison of the genome and those of three other species from Tetraclitidae shows that gene arrangement is identical, indicating that the mitochondrial gene order is highly conserved in the family. Moreover, in comparison with the pancrustacean ground pattern, the four species of Tetraclitidae share three large conserved gene blocks. Phylogenetic analysis based on 13 mitochondrial PCGs shows that *Chelonbia testudinaria* (Coronulidae) clusters with the four species of Tetraclitidae. Within Tetraclitidae, *T. serrata* clusters with *T. japonica*, and the two grouped with *T. rufotincta* with high support (BP = 100), with *T. divisa* as the most distantly related species (BP = 100).

Barnacles of the genus *Tetraclita* are common intertidal inhabitants of tropical and sub-tropical waters ( Newman and Ross 1976; Tsang et al. 2012). They are often the major space occupiers on the shore, playing important roles in the filter-feeding food chain and as foundation species affecting the structure and dynamics of intertidal communities (Barnes 2000). *Tetraclita rufotincta* Pilsbry 1916 is a common intertidal barnacle in the western Indian Ocean and east Africa (Achituv et al. 1997; Chan et al. 2009).

The work presents the complete mitochondrial genome of *T. rufotincta*. A specimen of *T. rufotincta* was obtained from the coastline in the Red Sea in Eilat, Israel, which is stored at the Biodiversity Research Museum, Academia Sinica, Taiwan. The mitochondrial genome encodes a set of 37 typical metazoan mitochondrial genes, including 13 protein-coding genes (PCGs), two ribosomal RNAs (rRNAs) genes, and 22 transfer RNAs (tRNAs) genes (GenBank accession number: KY865100). Four PCGs and two rRNAs are encoded on the light strand (*nd1*, *nd4*, *nd4L*, and *nd5*), while the other nine PCGs are located on the heavy strand.

In all 15 sessile barnacle mitochondrial genomes reported, the numbers of amino acids in four of the PCGs (*cox2*, *cox3*, *nd3*, *atp8*) are identical. Yet there are gene length variations in the remaining nine PCGs. The A + T composition of the first and second codon positions in the 13 PCGs of *T. rufotincta* is 49.3% and 66.3%, respectively, but that of the third codon positions elevates to 77.4%, which is lower than values from the other sessile barnacles (Shen et al. 2015, 2016).

The gene arrangement of *T. rufotincta* is identical to the other three mitochondrial genomes available from Tetraclitidae (*T. divisa*, *T. serrata*, and *T. japonica*). Compared with the pancrustacean ground pattern, the four species from Tetraclitidae share three large conserved gene blocks (*trnM*- *nd2*- *trnW*- *cox1*- *trnL_2_*- *cox2*- *trnD*- *atp8*- *atp6*- *cox3*- *trnG*- *nd3*- *trnR*- *trnN*- *trnA*- *trnE*- *trnS_1_*, *trnT*- *nd6*- *cob*- *trnS_2_*, and *nd1*- *trnL_1_*- *lrRNA*- *trnV*- *srRNA*) (genes encoded on the light strand are underlined). There are large variations in gene rearrangement among the Sessilia barnacles (Tsang et al. 2017), but the four species from Tetraclitidae have the same gene order, indicating that it is highly conserved within the family.

To investigate the phylogenetic position of the Sessilia and the relationships within this order, phylogenetic trees are constructed based on nucleotide sequences of 13 PCGs from 19 complete mitochondrial genomes of Maxillopoda using phyML (http://www.atgc-montpellier.fr/phyml/). In the tree (Figure 1), *Chelonbia testudinaria* (Coronulidae) clusters with the four species from Tetraclitidae. Within Tetraclitidae, *T. serrata* clusters with *T. japonica*, and the two are grouped with *T. rufotincta* with high support (BP = 100), with *T. divisa* being the most distantly related taxon (BP = 100). Tsang et al. (2015) provide molecular evidence suggesting that the superfamily Tetraclitoidea is monophyletic, while Tetraclitidae is not monophyletic. Thus, taxon coverage of the mitochondrial genome data from the family Tetraclitidae should be increased in order to address the issues on the monophyly of Tetraclitidae and the phylogenetic relationships within the family.

**Figure 1.**
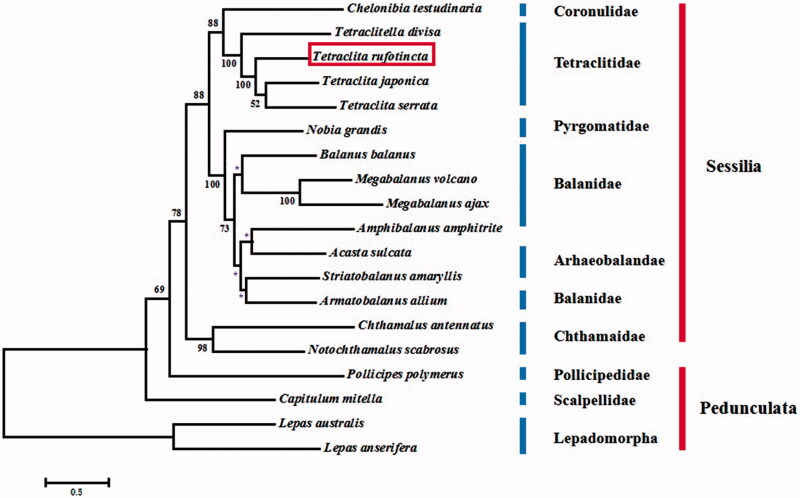
Maximum-likelihood phylogenetic tree based on 13 PCGs nucleotide acid sequences of *T. rufotincta* and 18 other Cirripedia species. Nodal supports are denoted on the corresponding branches for a bootstrap value >50% for ML, while * represents the value ≤50%.

## References

[CIT0001] Achituv Y, Brickner I, Erez J. 1997 Stable carbon isotope ratios in Red Sea barnacles (Cirripedia) as an indicator of their food source. Mar Biol. 130:243–247.

[CIT0002] Barnes M. 2000 The use of intertidal barnacle shells. Oceanogr Mar Biol. 38:157–187.

[CIT0003] Chan BKK, Hsu CH, Tsai PC. 2009 Morphology and distribution of the acorn barnacle *Tetraclita reni* nom. nov. (Crustacea: Cirripedia) in Madagascar and adjacent waters. Zootaxa. 2019:57–68.

[CIT0004] Newman WA, Ross A. 1976 Revision of the balanomorph barnacles; including a catalogue of the species. Memoir San Diego Soc Nat Hist. 9:1–108.

[CIT0005] Shen X, Chu KH, Chan BKK, Tsang LM. 2016 The complete mitochondrial genome of the fire coral-inhabiting barnacle *Megabalanus ajax* (Sessilia: Balanidae): gene rearrangements and atypical gene content. Mitochondrial DNA. 27:1173–1174. 25050875 10.3109/19401736.2014.936424

[CIT0006] Shen X, Tsang LM, Chu KH, Achituv Y, Chan BKK. 2015 Mitochondrial genome of the intertidal acorn barnacle *Tetraclita serrata* Darwin, 1854 (Crustacea: Sessilia): gene order comparison and phylogenetic consideration within Sessilia. Mar Genomics. 22:63–69. 25907711 10.1016/j.margen.2015.04.004

[CIT0007] Tsang LM, Achituv Y, Chu KH, Chan BKK. 2012 Zoogeography of intertidal communities in the west Indian Ocean as determined by ocean circulation systems: patterns from the *Tetraclita* barnacles. PLoS One. 7:e45120. 23024801 10.1371/journal.pone.0045120 PMC3443201

[CIT0008] Tsang LM, Chu KH, Achituv Y, Chan BKK. 2015 Molecular phylogeny of the acorn barnacle family Tetraclitidae (Cirripedia: Balanomorpha: Tetraclitoidea): validity of shell morphology and arthropodal characteristics in the systematics of tetraclitid barnacles. Mol Phylogenet Evol. 82:324–329. 25263422 10.1016/j.ympev.2014.09.015

[CIT0009] Tsang LM, Shen X, Cheang CC, Chu KH, Chan BKK. 2017 Gene rearrangement and sequence analysis of mitogenomes suggest polyphyly of archaeobalanid and balanid barnacles (Cirripedia: Balanomorpha). Zool Scripta. 46:729–739.

